# Implant waste and associated costs in trauma and orthopaedic surgery: a systematic review

**DOI:** 10.1007/s00264-024-06397-w

**Published:** 2025-01-04

**Authors:** Fizza Ali, Muhayman Sadiq, Yasser Al Omran, Thomas Lewis, Peter Bates, Ruben Doyle, Omar Musbahi

**Affiliations:** 1 GKT School of Medicine, Guys Campus, London, SE1 1UL UK; 2 Royal Free Hospital, Pond St., London, NW3 2QG UK; 3 King’s College Hospital NHS Foundation Trust, Denmark Hill, London, SE5 9RS UK; 4 Royal London Hospital, Barts Health NHS Trust, London, UK; 5 Department of Engineering, Imperial College London, London, SW7 2AZ UK; 6 MSk Lab, Imperial College London, London, W12 0BZ UK; 7 Imperial College Healthcare NHS Trust, Praed Street, London, W2 1NY UK

**Keywords:** Implant waste, Metalwork, Trauma and orthopaedic surgery, T&O, Hospital costs

## Abstract

**Purpose:**

Trauma and orthopaedic (T&O) surgery relies on medical implants and materials, often resulting in metalwork wastage (prosthesis, screws, nails, and plates). This places an economic strain on healthcare services and the environment. Our primary outcome is to quantify the implant wastage across the literature, and secondarily investigate the associated costs in this specialty.

**Methods:**

A literature search of three databases (Scopus, PubMed and Embase) was performed using MeSH terms relating to “implant waste” and “trauma and orthopaedic surgery”, from January 1980 to November 2023. We included any observational studies that reported patients undergoing T&O surgery, where the wastage or associated costs was reported.

**Results:**

Our search returned 2,145 articles, of which 15 met the final inclusion criteria, encompassing 26,832 procedures. Nine studies reported the extent and cost of waste, six reported the weight of waste and ten concurrently reported the cost. Implant waste events occurred in up to 30% of all T&O procedures, being the most likely to occur in fracture fixation, and cost hospitals between $4,130 and $189,628.41 annually. Screws were the most wasted material, followed by plates and nails. Up to 95% of waste events were caused by human factors.

**Conclusion:**

Despite the limited number of studies, there is an economic burden and environmental footprint in T&O surgery services. The main factors contributing to the waste was human error, and contamination. Further research is required to determine methods of mitigating and limiting implant waste in T&O Surgery.

**Supplementary Information:**

The online version contains supplementary material available at 10.1007/s00264-024-06397-w.

## Introduction

According to the World Health Organisation (WHO), musculoskeletal injuries accounted for 16% of the global disease burden in 2015 [1]. In 2022, 18,577,953 Trauma and Orthopaedic surgery (T&O) procedures were performed in the United States (US) and just over 350,000 in the United Kingdom (UK) [2, 3]. Osteoporosis-related fragility fractures and osteoarthritis are two of the most common presentations requiring surgical intervention (fracture fixation and joint replacement respectively), which is often the single most effective treatment [4, 5]. The Global Burden of Disease study looked at the global prevalence of fractures across 204 countries from 1990 to 2019, and reported 178 million fractures in 2019, a 33.4% increase since 1990 [6]. A report of fragility fractures in the European Union saw 3.5 million fragility fractures in 2010, expected to rise to 4.5 million annually in 2025 [7]. Demand for these procedures, amongst others, are projected to increase due to a globally ageing population and lifestyle changes increasing life expectancy.

T&O is a material-heavy specialty due to its reliance on metal implants, such as screws, nails, plates and prosthesis. In 2022, the US orthopaedic implant industry was valued at $43.13 billion and is estimated to grow to $64.27 billion by 2030. However, it is theorised due to the high amount of materials and equipment required in orthopaedics, there is a growing concern regarding the waste, sustainability and environmental impact in T&O surgery [8]. A review by Robinson et al. found medical devices and single-use items as being the key contributor to hospital carbon footprint across five studies [9]. Screws are one of the most common T&O implant used and its market size is predicted to increase worldwide by up to 7.4% spurred by factors including an ageing population and technological advancements [10]. It is also consequently the most frequently wasted implant [11]. Whilst screws were the most readily wasted implant, joint replacements, particularly total hip arthroplasty (THA) and total knee arthroplasty (TKA) have also been shown to contribute the highest waste-generating procedures in T&O [12]. The demand of these two procedures are projected to increase by 123% and 605% respectively by 2030 [13]. Therefore, sustainable hospital pathways for intraoperative waste production need to be considered to minimise the forthcoming environmental impact of these procedures.

Healthcare providers, researchers and government policies recognise T&O as a key contributor to the hospital carbon footprint and are attempting to address the economic and environmental impact of implant waste in T&O surgery [8]. This has reported promising results in T&O, such as by organising waste segregation and preventing inappropriate disposal [14], minimalizing equipment included in disposable surgical packs to only essentials [15], and the ‘Getting It Right The First Time (GIRFT)’ scheme in the United Kingdom which has made sustainability in orthopaedics a key target over the next decade [16]. The UK’s National Health Service (NHS) hopes these interventions will contribute to their goal of being ‘net zero’ by 2040 [17].

To our knowledge there is currently no review that has aimed to quantify the amount of waste and the environmental impact of T&O surgery. This systematic review aims to quantify and evaluate the amount, cost and environmental impact of implant waste in T&O surgery reported across the literature.

## Methods

### Study design

This systematic review collated all available literature meeting the inclusion criteria to form a narrative synthesis. It was prospectively registered on PROSPERO (CRD42023443444) and followed ‘Preferred Reporting Items for Systematic Reviews and Meta-Analyses’ (PRISMA) reporting guidelines [18].

### Search strategy

This study was conducted between 1 November 2023 and 31 March 2024. A comprehensive literature search of scientific, original articles across three databases (Scopus, PubMed and Embase) was undertaken from January 1966 (beginning of records) to November 2023 by FA and MS. Our search strategy included MeSH terms and keywords encompassing implant waste and T&O surgery (supplementary material, item A and item B).

### Inclusion and exclusion criteria

Studies were deemed eligible for inclusion if the participants (of any age, ethnicity, and gender) underwent a T&O surgery requiring the use of prosthesis, plates, screws or nails and the number of this was reported in the study. For the purpose of this study, an orthopaedic “Implant” referred to a medical device inserted into the body to maintain, improve, replace or re-establish function of that joint or bone [19]. This may be due to traumatic damage or deformity of the natural structure. The implants of interest in this study include screws, nails, plates and prosthesis. We included all elective and trauma procedures taking place in a T&O theatre reported as observational (prospective and retrospective, single- or multi-centre) cohort studies or randomised control trials. We excluded case reports.

### Intervention

All emergency and elective T&O procedures requiring implants or associated materials were investigated. The type of procedure was recorded to allow for identification of patterns in the type of procedure or sub-specialty.

### Data extraction

We collected data related to publication information (author(s), publication year, country of study), procedure characteristics (number and type of procedures), implant usage (total number of implants used and wasted, type implant), associated cost of implant wastage and further study findings. Two reviewers (FA and MS) independently screened each study for inclusion criteria, blinded to the others’ decision. Any difference of opinion was resolved by discussion and agreement with a third author (OM, TL). The final studies for inclusion underwent data extraction.

### Outcome

The primary outcome of this study was to report the proportion and amount of implant and associated waste in T&O surgery, and the hospital costs attributed to this wastage. Our secondary outcome was to quantify the hospital cost of wasted implants. Where there as available data, we also investigated causative factors of implant waste.

### Risk of Bias assessment

The study quality assessment and critical appraisal was assessed using the ‘Methodological Index for Non-Randomised Studies’ (MINORS) criteria [20]. Two reviewers (FA and MS) scored each study out of a maximum 16 points for non-comparative studies, classifying a poor quality ( = < 8), moderate quality (9–14) or good quality (15–16) study. Comparative studies had a maximum score of 24 and classification cut-offs at = < 14, 15–22 and 23–24 respectively. Where there was a discrepancy, a third reviewer (OM) helped to resolve any disagreements.

## Results

**Fig. 1:**
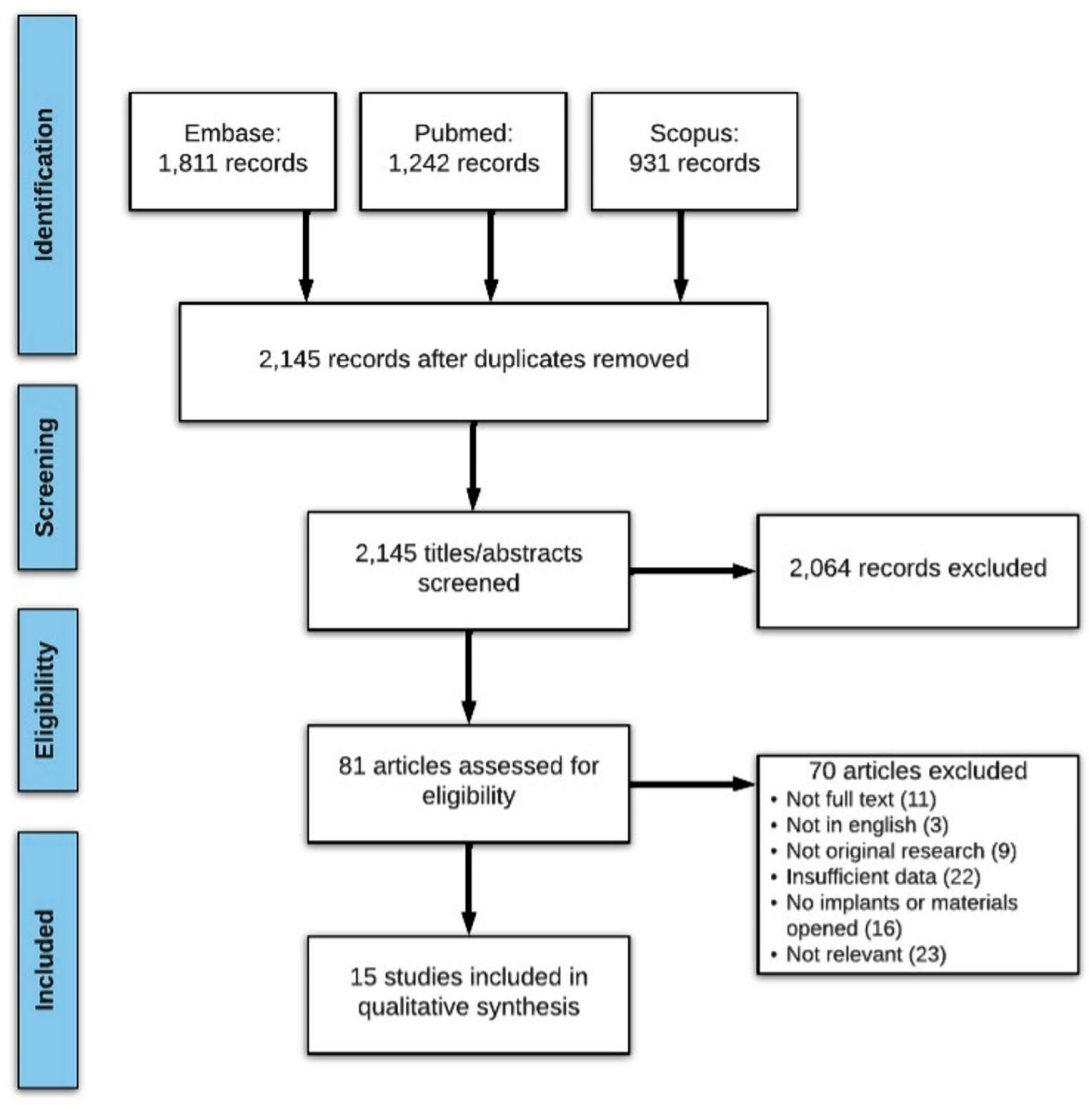
PRISMA flow diagram

Our search returned 2,145 articles which were screened for title and abstract, followed by full-text screening. Fifteen articles fulfilled all inclusion criteria (see PRISMA, Fig. 1), encompassing 26,832 procedures. Nine reported the extent of metalwork waste (Table 1) of which five reported reasons for waste (Table 2), and six as weight of wastage (Table 3). Cost of wastage was concurrently reported in 10 papers across these two categories.

**Table 1:** Summary of final studies included in this systematic review. Below are nine studies reporting the extent of implant waste and associated costs

Author, year; Country	Study design	Primary outcome	Study duration (years)	Procedure type	Number procedures with waste/ total procedures	Number of wasted implants / total implants	Details of waste	Cost of wasted implants	Details of cost
Ast et al., 2014; US	Retrospective observational cohort	Percentage of implant waste and cost analysis following e-Label intervention	1	TKA	NR/1450 (pre-intervention), NR/244 (post-intervention)	83/NR (pre-intervention), 2/NR (post-intervention)	After introducing an intraoperative e-Label intervention, implant waste reduced from 5.7–0.2%	$224,331 (pre-intervention), $4,130 (post-intervention)	Annualised cost saving of $217,251 with the intervention
Epstein et al., 2015; US	Retrospective observational, single-centre cohort	Cost and reasons of waste following a 2-year educational intervention	2.6 (31 months)	Lumbar spinal fusion	NR	NR	Surgeon-related factors remained the major cause for implant waste	$164,477	Cost of waste was reduced by 64.9% over 2 years
Jayakumar et al., 2019; UK	Retrospective observational single-centre cohort study	Implant wastage in T&O surgery and associated costs	1	Trauma	142/565 (25.1%)	227/NR	91% of waste was screws were wasted (206). 142 (25.1%) of procedures were affected, 65% of which were lower-limb trauma	£8,377.25 (2% of total implant budget)	47.6% total cost was due to intramedullary nail waste. Nail waste cost £3,986.90 in total, screws £2,432.14 and plates £1,958.22.
Laurut et al., 2019; France	Retrospective observational	Percentage of wasted implants and mean cost	1	T&O	NR	1995/29,073 (6.9%)	Screws had the highest wastage in trauma surgery (1334/14,642, 9.1%). Trauma had significantly more wasted implants than orthopaedic surgery, although individual trauma implants cost less	€179,193 (4.8% of total implant budget)	Total cost of all implants (wasted and used) €3,761,180
Payne et al., 2015; US	Retrospective economic analysis	Identifying the types of implants wasted, incidence and cost of waste	1	T&O	NR/8954	1072/8954 (12.0%)	In trauma surgery specifically, 157/517 (30.4%) implants were wasted	$634,668 (1.8% total cost of implants)	Total implant cost was $34,340,607. Trauma total cost of implants was $1,224,809 and wastage was $42,888
Pfefferie et al., 2015; US	Single-centre interventional	Cost per waste event, number of waste events in operations before and after cost-awareness intervention	1	THAs, TKAs and shoulder arthroplasties	60/3973 (1.5%)	NR	NR	$178,207.63	Pre-intervention: cost of waste 25/1662 THA and TKA averaged $4,878.32 per procedure, 7/206 shoulder arthroplasties averaged $626.46
Soroceanu et al., 2011; US	Prospective observational	The impact of an educational programme on the incidence of waste and associated costs	1.25 (15 months)	Spinal surgery	263/1304 (20.2%)	312/739 (42.2%)	Waste dropped from 20.2–10.3% post-intervention	$234,868. (Annual estimate of $126,722,000)	$186,973.65 wasted due to ‘surgeon changed mind.’
Zywiel et al., 2009; US	Retrospective observational, multi-centre cohort	Extent and cost of trauma implant waste	1.5 (18 months)	Trauma surgery	37/6531 (5.7%)	NR	95% (35/37) waste events due to OR staff or surgeon error	$27,653	Mean cost per waste event was $747.38. Annual cost of $18,435.33.
Zywiel et al., 2010; US	Retrospective observational, multi-centre cohort	Extent and cost of THA and TKA implant waste	1.5 (18 months)	THAs, TKAs	79/3443 (2%)	82/NR	Waste events in 44/1,542 THA procedures (3%) and 35/1901 TKAs (2%). 80% waste events due to OR staff or surgeon error	$163,943.02 (THA $91,999.52; TKA $71,943.50)	Mean cost per waste event was $2,075.23. Annual cost of $109,295.35 ($61,333.01 from THA, $47,962.33 from TKA). Average cost of wasted component was more expensive in THAs ($2,090.90) than TKAs ($2,055.53)

Abbreviations: NR, not reported; OR, operating room; ORIF, open reduction internal fixation; T&O, trauma and orthopaedics; THA, total hip arthroplasty; TKA, total knee arthroplasty; UK, United Kingdom; US, United States

**Table 2:** Five studies reporting reasons for implant waste

		Reasons for implant waste, % (*n*/total waste)				
		Ast et al., 2014	Epstein et al., 2015	Soroceanu et al., 2011	Zywiel et al., 2009	Zywiel et al., 2010
Type of surgery		TKA	Spinal surgery	Spinal surgery	Trauma surgery	TKA, THA
Length of study (months)		12	31	15	18	18
Number of procedures investigated (n)		1450		1304	6531	1901 TKAs; 1542 THAs
Total number of implants wasted (n)		83	190	739	37	35 TKAs with waste; 44 THAs with waste
Human factors for waste		83 (68/83)	95.4 (181/190)	74.29 (549/739)	95 (35/37)	TKA waste events, 66; THA waste events, 80
	Size selection indecision	55.4 (46/83)	11.6 (22/190)	44.11 (326/739)		
	Surgeon factors		70 (133/190)			
	Mistakenly opened implant	8.4 (7/83)	1.1 (2/190)	3.24 (24/739)		
	Opened but not implanted		7.4 (14/190)			
	Contamination	7.2 (6/83)	4.2 (8/190)	26.93 (199/739)		
	Defective or damaged during use	12.0 (10/83)	1.1 (2/190)			
Non-human factors for waste			4.7 (9/190)	25.71 (190/739)		TKA waste events, 34; THA waste events, 20
	Case cancellation			15.83 (117/739)		
	Equipment failure			4.06 (30/739)		
	Other/no reason stated	14.5 (12/83)		5.82 (43/739)		

Abbreviations: THA, total hip arthroplasty; TKA, total knee arthroplasty

**Table 3:** Summary of final studies included in this systematic review. Below are six studies reporting the weight of implant waste

Author, Year; Country	Study Design	Primary Outcome	Duration of Study	Number of T&O procedures	Procedure type	Non-recyclable/Total Waste (kg)	Recyclable/Total waste (Kg)	Details of waste
Hennessy et al., 2021; Ireland	Prospective observational, single-centre cohort	Weight of implant packaging waste	1 year	209	ORIF ankle, humerus and clavicle; hemiarthroplasty	4.791/NR	NR	2.768/4.791 kg was cardboard waste, 2.023/4.791 kg was plastic waste. In one ankle ORIF, 110 g (52%) of packaging waste was from screws
Huet et al., 2022; France	Prospective observational, single-centre cohort	Cost comparison of procedures using single-use and reusable instruments	16 months	46	Distal radius fracture	NR/13.2	NR/13.2	The cost of using single-use implants and equipment (waste management, steralisation, staff) was less than re-usable equipment, but waste mangement was overall more expensive (€6500 vs. €6100)
Kooner et al., 2020; Canada	Multi-centre, prospective, observational cohort study	Quantifying the amount of recyclable and non-recyclable waste in 6 sub-specialties.	30 days	55	Arthroplasty (14), trauma (10), sports (10), upper limb (12), paediatrics (5), foot and ankle (4)	239/341 (70.1%)	93.4/341 (27.4%)	Average of 21,928.2 kg intraoperative non-recyclable waste and 1,890.9 kg recyclable waste over 1 month.
Pegg et al., 2022; United Kingdom	Single-centre, prospective, observational cohort study	Amount of recyclable and non-recyclable waste	2 days	3	THA (3)	28.5/32.5 (87.7%)	4.2/32.5 (12.9%)	Average waste per procedure was 10.9 kg.
Rammelkamp et al., 2021; United States	Single-centre, prospective, interventional study	Quantifying the amount of waste produced in an OR suite	10 days	23	TKA (14), THA (3), TSA (6)	304.1/337.4 (90.1%)	33.3/337.4 (9.9%)	TKA produced the most waste per procedure (15.6 kg), followed by TSA (13.kg) and THA (41.4 kg).
Southorn et al., 2013; United Kingdom	Multi-centre, prospective, observational cohort study	Amount of waste produced by arthroplasty	14 days	32	TKA (14), THA (18)	178.6/380.2 (47.0%)	NR/380.2	Total waste of 162.4 kg generated from TKA and 217.8 kg from THA

Abbreviations: FJI, facet joint injection; NR, not reported; OR, operating room; ORIF, open reduction internal fixation; T&O, trauma and orthopaedics; THA, total hip arthroplasty; TKA, total knee arthroplasty; TSA, total shoulder arthroplasty

### Extent of metalwork waste

Across nine studies, implant waste events occurred in 0.8-30% of all 26,464 procedures included. Two studies focused on trauma surgery [11, 21], five on elective orthopaedic procedures (TKA, THA, spinal surgery) [22–26] and two looked at both [27, 28].

Trauma surgery saw greater rates of implant wastage than orthopaedic surgery. This was seen by a single-centre comparison over a 12-month period, with a wastage rate of 1135/1995(7.4%) in trauma compared to 860/1995(6.3%) in orthopaedic surgery [27]. The same study concluded that fracture fixation produced the greatest quantities of individual metalwork waste out of all investigated T&O procedures (8.3% of all fracture fixation implants were wasted), followed by ligament reconstruction(7.1%), THA(4.9%), shoulder arthroplasty(4.3%) and TKA(2.4%) [27]. A US-based study looking at 8,954 cases over 12 months saw waste occurring in up to 30% of all trauma procedures(157/517) and 14.6% of all T&O procedures(157/1072), which was followed by foot & ankle (23%, 67/297) [28].

Screws were the most likely implant to be wasted in trauma surgery. This was seen in the study by Laurut et al., with a wastage rate of 9.1% (1334 wasted out of 14,642 opened) [27] and also Payne et al. where screws, particularly cortical, were wasted most in trauma procedures [28]. Jayakumar et al. investigated waste in trauma surgery where 91% of all wasted implants were screws (207/227) [11].

In elective orthopaedic surgery, joint arthroplasties were the most thoroughly investigated and concluded that THAs are more likely to generate waste than TKAs. The incidence of a waste event in THA surgery was 9% (100/1076), compared to 4% (42/1003) in TKAs [28]. Zywiel et al. saw similar a waste incidence of 3% in THAs (waste events in 44/1,542 procedures) and 2% in TKAs(35/1901), also based in the US [26]. The most common implant to be wasted in THAs were acetabular cups (6.4%, 69/1081) and inserts (6.6%, 41/623); knee and shoulder arthroplasty saw inserts as the most wasted implant (5.4%, 18/335 and 6.7%, 5/75 respectively) [27].

Five studies explored reasons for implant waste, with up to 95% of waste events being attributable to human error of the surgeon or operating room staff (see Table 2) [21–23, 25, 26].

### Cost of waste

Ten out of fifteen papers reported the cost of waste, which ranged between $4,130 and $189,628.41 annually, depending on study variables such as country, healthcare service provision (government funded or private), and hospital location (e.g., tertiary care, district hospital) [11, 21–29].

A single-centre study based in the UK, the total cost of implant wastage amounted to £8,377.25 over 12 months., Individual screws had the lowest unit costs (£61.63), whilst intramedullary nails had the most expensive unit costs (£607) [11]. Nails were wasted the least, however high unit costs drove up the overall cost of wastage if wasted, ultimately contributing to 47.6% of the overall cost of wasted metalwork (48%, £3,986.90), followed by screw (29%, £2,432.14) and plate waste (23%, £1,958.22) [11]. Fracture fixation was the most wasteful procedure and subsequently one of the most costly, accounting for up to 29% of the total cost of waste [27].

Pfefferie et al. looked at cost implications of joint arthroplasty in the US before and after a cost awareness intervention, where the average cost of waste from a THA or TKA procedure increased ($2,555.13 to $4,878.32 per event) whilst shoulder arthroplasties decreased ($1,149.92 to $626.46) [24]. The electronic intervention proposed by Ast et al. to screen TKA implants before opening yielded a cost reduction of implant waste from $224,331 to $4,130 over seven months, an annual cost saving of $217,251 [22]. A two-year educational intervention for spinal orthopaedic surgeons in the US yielded a significant reduction in the extent and cost of waste, from $92,688 to $39,284– a 64.7% reduction [23]. This reduction continued for another nine months down to $32,505, a further 61% reduction [23].

### Weight of waste

Six studies reported the physical weight of waste associated intraoperative implant materials (Table 3) [29–33]. Across 368 procedures, there was a total wastage of 433.18 kg out of which 313.2 kg (72.3%) was non-recyclable, to include implants. Joint arthroplasty was the most wasteful sub-speciality for non-recyclable waste (5.8 kg per case) and recyclable waste, followed by trauma (4.3 kg) and sports surgery (4.0 kg) [30, 32, 33].

Out of six T&O sub-specialties, joint arthroplasty exhibited the greatest total amounts of recyclable, non-recyclable and biological waste than others over a one-month audit [30]. Paediatrics had the greatest proportion of recyclable waste: 42.6% (2,158/5,604 kg) of its waste was recyclable, followed by 33% (2,955.7/8,779.3 kg) for joint arthroplasty [30]. Rammelkamp et al. conducted a five day audit where the average waste generated from TKA was greater than THA and shoulder arthroplasties (Table 3) [32]. This finding differed from a UK-based study which found the average waste generated from 18 THAs (12.1 kg) was greater than the average waste from 14 TKAs (11.6 kg) [33]. The authors concluded that appropriate waste segregation and recycling would reduce the operative carbon footprint by an estimated 75% [33].

### Risk of bias assessment

According to MINORS criteria, three studies were of good methodological quality [21, 26, 27] and 12 were of moderate quality (see Appendix A). There were five comparative studies, all of which were of moderate quality [22–25, 29]. All studies had a clearly stated aim and most had appropriate inclusion criteria with sufficient follow-up period to identify significant results. The greatest bias was introduced from absent prospective study size calculation across seven studies and unjustified end points in seven studies. Four out of five comparative studies used historical controls which introduced bias.

## Discussion

In this study, we quantified implant waste and associated costs in T&O procedures. Trauma represented the largest proportion of waste compared to elective orthopaedic procedures and subsequent greatest costs to each healthcare system in the studies – up to 30% of all trauma surgeries reported waste events [28]. Three studies concluded that screws accounted for the greatest extent of wastage, followed by nails and plates [11, 27, 34]. Intramedullary nails were least likely to be wasted but were the biggest cost burden as they are an extremely high-value implant [11]. Five papers reported the impact of human factors on the extent of waste, showing that up to 95% of waste was caused by surgeons or theatre staff either changing their mind about the size, opening the incorrect size, damaging implants or contamination [21–23, 25, 26].

Our findings showed human error was the leading cause for implant waste [21–23, 25, 26]. In a survey, 85% of orthopaedic surgeons were concerned about pollution arising from the industry, and most (61%) orthopaedic surgeons ranked ‘cost’ as an important factor when requesting single-use theatre supplies [35], however 54–67% of orthopaedic surgeons were found to underestimate theatre and implant costs [35, 36]. Improving awareness of implant costs amongst staff is necessary, and results have already shown it to be an effective intervention. Soroceanu et al., found that a ten month educational programme to improve cost-awareness amongst orthopaedic surgeons led to a drop in waste of 49%, from 20.2–10.3% [25]. Another intervention involved publicising theatre wastage caused by individual orthopaedic surgeons in a staff room, which prompted increased self-awareness and a non-significant reduction of waste events from 1.50–1.11% [24].

Ast et al. looked at TKA implant waste 12 months before and 7 months after implementing a computer-based intervention, designed to reduce human error and consequently reduce waste by screening implants before opening through an eight point checklist, including implant size, side, expiration date and manufacturer. This showed a significant reduction of arthroplasty waste from one wasted implant in 5.7% of procedures (83 waste items per 1450 TKAs) to 0.8% (2/255) over 7 months, and an annual cost saving of $75,685.95 [22]. Wrong-site or side surgery has been seen in orthopaedic cases [37], as well as difficulty in reading implant labels and therefore opening incorrect implants [38], however initiatives have been launched to include labelling the site of surgery [39], and following labelling recommendations to make the implant packaging easier to read [40].

Trauma surgery saw high rates of implant waste and associated costs, particularly fracture fixation; elective orthopaedics had lower rates of waste but individual implants were expensive, driving up the overall cost if an implant was wasted. This places an economical strain on hospitals, in addition to cost of metalwork disposal and theatre running costs, which is a challenge with trusts already facing financial pressures [21, 23, 27]. This non-recyclable waste also has environmental implications from pollutants released during the production and disposal process [8]. Many surgical specialties are striving towards a greener, more sustainable operating room; most implant waste is caused by surgeon intraoperative decision making or user case so targeting this would be the first step towards greener practice.

## Future directions

Future directions for sustainable T&O practice could aim to integrate technologies to minimise the impact of intraoperative human error, thereby enhancing surgical precision and safety.

Technology may reduce the risk of surgical errors and enhances patient safety. Pre-operative planning and smart implantable T&O devices are equipped with sensors and wireless connectivity, allowing real-time intraoperative feedback such as implant position, biomechanical forces, and physiological parameters, enabling surgeons to make informed decisions during surgery and ensuring optimal implant placement [41]. Robotic-assisted T&O surgery can also enhance intraoperative accuracy by assisting surgeons in performing tasks with sub-millimetre accuracy and reproducibility, reducing variability and minimizing the risk of human error [42]. By integrating robotics into T&O, healthcare providers can improve patient outcomes, reduce implant waste, and optimize resource utilization in the operating room.

Further research and development in this area are essential to realizing the full potential of these technologies and advancing the field of T&O surgery towards a greener, more efficient future.

## Limitations

A key limitation to our study was the challenge posed by the lack of matched data across the included papers. Discrepancies in the information provided by each study along with disproportionate patient groups, hindered the ability to accurately calculate summary measures. This variability in data presentation and patient characteristics made it difficult to conduct a meta-analysis of proportions. The heterogeneity of data and potential reporting bias further compounded these challenges. Implementing such standards would not only facilitate more accurate comparisons and analyses across studies but also enhance the reproducibility and reliability of findings in the field of T&O surgery.

## Conclusion

Effectively managing the soaring costs of implant materials while safeguarding patient safety presents a formidable challenge across T&O departments. This systematic review demonstrates the significant waste documented in the literature within this domain, along with its substantial financial implications. Notably, T&O procedures incur heightened expenses due to the augmented volume of waste, primarily attributed to human factors such as surgical and staff practices. Despite efforts to address these factors, interventions must extend comprehensively across all subspecialties of T&O to yield meaningful results. This calls for a concerted effort to scale up interventions aimed at mitigating human error and optimizing resource utilization throughout the entire spectrum of T&O care, ensuring both fiscal responsibility and patient well-being.

## Appendix A: Risk of bias assessment using the Methodological Index for Non-Randomised Studies (MINORS) criteria

**Table:** 

	Clearly Stated Aim	Inclusion of Consecutive Patients	Prospective Collection of Data	Endpoints Appropriate to the Aim of the Study	Unbiased Assessment of the Study Endpoint	Follow-Up Period Appropriate to the Aim of the Study	Loss to Follow Up Less Than 5%	Prospective Calculation of Study Size	An adequate control group	Contemporary groups	Baseline equivalence of groups	Adequate statistical analyses	Total	Grade
Ast et al., 2014*	2	1	2	2	2	2	2	0	2	0	1	0	16	moderate
Epstein et al., 2015*	2	0	2	2	1	2	2	0	1	0	2	0	14	moderate
Hennessy et al., 2021	2	2	0	2	0	2	2	0					10	moderate
Huet et al., 2022*	2	2	2	2	2	2	1	2	1	2	2	2	22	moderate
Jayakumar et al., 2019	2	2	0	2	2	2	2	0					12	moderate
Kooner et al., 2020	2	0	2	1	0	1	2	2					10	moderate
Laurut et al., 2019	2	2	1	2	2	2	2	2					15	good
Payne et al., 2015	2	2	0	2	2	2	2	2					14	moderate
Pegg et al., 2022	2	2	2	1	0	1	2	0					10	moderate
Pfefferie et al., 2015*	2	2	0	2	2	2	2	2	0	0	2	2	18	moderate
Rammelkamp et al., 2021	2	2	2	1	0	1	2	0					10	moderate
Soroceanu et al., 2011*	2	2	2	2	0	2	2	2	0	0	2	2	18	moderate
Southorn et al., 2023	2	2	2	2	1	2	2	0					13	moderate
Zywiel et al., 2009	2	2	2	2	2	2	2	2					16	good
Zywiel et al., 2010	2	2	2	2	2	2	2	2					16	good

* only apply to comparative studies. Maximum score of 16 for non-comparative studies and 24 for comparative studies

## Electronic supplementary material

Below is the link to the electronic supplementary material.

Supplementary Material 1

## References

[1] WHO (2008) The global burden of disease: 2004 update. World Health Organisation. https://www.who.int/publications/i/item/9789241563710. Accessed 3 March 2024

[2] British Orthopaedic Association B (2022) Policy Position Statements. British Orthopaedic Association, BOA. https://www.boa.ac.uk/resource-library.html?information_type=policy-position-statements. Accessed 4 March 2024

[3] GlobalData (2023) United States (US) Orthopedic Procedures Count by Segments and Forecast to 2030. GlobalData. https://www.globaldata.com/store/report/usa-orthopedic-procedures-analysis/. Accessed 5 March 2024

[4] Brumat P, Kunšič O, Novak S, Slokar U, Pšenica J, Topolovec M, Mihalič R, Trebše R (2022) The Surgical treatment of Osteoarthritis. Life (Basel). 10.3390/life12070982 35888072 10.3390/life12070982 PMC9319328

[5] van Oostwaard M (2018) Osteoporosis and the nature of fragility fracture: an overview. In: Hertz K, Santy-Tomlinson J (eds) Fragility fracture nursing: Holistic Care and management of the Orthogeriatric patient, 1st edn. Springer International Publishing, Cham, pp 1–13

[6] Disease GB (2021) Global, regional, and national burden of bone fractures in 204 countries and territories, 1990–2019: a systematic analysis from the global burden of Disease Study 2019. 10.1016/s2666-7568(21)00172-0. Lancet Healthy Longev 10.1016/S2666-7568(21)00172-0 PMC8547262 34723233

[7] Hernlund E, Svedbom A, Ivergård M, Compston J, Cooper C, Stenmark J, McCloskey EV, Jönsson B, Kanis JA (2013) Osteoporosis in the European Union: medical management, epidemiology and economic burden. A report prepared in collaboration with the International Osteoporosis Foundation (IOF) and the European Federation of Pharmaceutical Industry Associations (EFPIA). Arch Osteoporos. 10.1007/s11657-013-0136-1 24113837 10.1007/s11657-013-0136-1 PMC3880487

[8] Engler ID, Curley AJ, Fu FH, Bilec MM (2022) Environmental sustainability in orthopaedic surgery. JAAOS -. J Am Acad Orthop Surg. 10.5435/jaaos-d-21-01254 10.5435/JAAOS-D-21-01254 35412500

[9] Robinson P, Surendran K, Lim S, Robinson M (2023) The carbon footprint of surgical operations: a systematic review update. Annals Royal Coll Surg Engl. 10.1308/rcsann.2023.0057 10.1308/rcsann.2023.0057 PMC10626532 37906978

[10] FutureMarketInsights (2021) Bone Screw System Market Outlook from 2024 to 2034. Future Market Insights. https://www.futuremarketinsights.com/reports/bone-screw-system-market. Accessed 7 March 2024

[11] Jayakumar N, Munuswamy S, Kulshreshtha R, Deshmukh S (2020) Implant wastage in orthopaedic trauma: a UK experience. Ann R Coll Surg Engl. 10.1308/rcsann.2019.0151 31660763 10.1308/rcsann.2019.0151 PMC7027411

[12] Phoon KM, Afzal I, Sochart DH, Asopa V, Gikas P, Kader D (2022) Environmental sustainability in orthopaedic surgery: a scoping review. Bone Jt Open. 10.1302/2633-1462.38.Bjo-2022-0067.R1 35965477 10.1302/2633-1462.38.BJO-2022-0067.R1 PMC9422904

[13] Kurtz S, Ong K, Lau E, Mowat F, Halpern M (2007) Projections of primary and revision hip and knee arthroplasty in the United States from 2005 to 2030. J Bone Joint Surg Am. 10.2106/jbjs.F.00222 17403800 10.2106/JBJS.F.00222

[14] Albert MG, Rothkopf DM (2015) Operating room waste reduction in plastic and hand surgery. Plast Surg (Oakv). 10.4172/plastic-surgery.1000941 26665137 10.4172/plastic-surgery.1000941 PMC4664137

[15] Thiel CL, Fiorin Carvalho R, Hess L, Tighe J, Laurence V, Bilec MM, Baratz M (2019) Minimal Custom Pack Design and Wide-Awake Hand Surgery: Reducing Waste and Spending in the Orthopedic Operating Room. Hand (N Y). 10.1177/1558944717743595 10.1177/1558944717743595 PMC6436127 29183168

[16] Briggs T (2015) A national review of adult elective orthopaedic services in England. Getting it right first time. British Orthopaedic Association. https://gettingitrightfirsttime.co.uk/wp-content/uploads/2018/07/GIRFT-National-Report-Mar15-Web.pdf. Accessed 12 March 2024

[17] NHS, NHS England (2022) Delivering a net zero NHS. https://www.england.nhs.uk/greenernhs/a-net-zero-nhs/#:~:text=On%201%20July%202022%2C%20the,trusts%20and%20integrated%20care%20boards. Accessed 12 March 2024

[18] Moher D, Liberati A, Tetzlaff J, Altman DG (2009) Preferred reporting items for systematic reviews and meta-analyses: the PRISMA statement. Ann Intern Med. 10.7326/0003-4819-151-4-200908180-00135 19622511 10.7326/0003-4819-151-4-200908180-00135

[19] WHO (2019) International Classification of Diseases ICD-10. World Health Organisation. https://www.who.int/standards/classifications/classification-of-diseases. Accessed 12 March 2024

[20] Slim K, Nini E, Forestier D, Kwiatkowski F, Panis Y, Chipponi J (2003) Methodological index for non-randomized studies (minors): development and validation of a new instrument. ANZ J Surg. 10.1046/j.1445-2197.2003.02748.x 12956787 10.1046/j.1445-2197.2003.02748.x

[21] Zywiel MG, Delanois RE, McGrath MS, Ulrich SD, Duncan JL, Mont MA (2009) Intraoperative waste of trauma implants: a cost burden to hospitals worth addressing? J Orthop Trauma. 10.1097/BOT.0b013e3181af69a6 19858979 10.1097/BOT.0b013e3181af69a6

[22] Ast MP, Mayman DJ, Su EP, Gonzalez Della Valle AM, Parks ML, Haas SB (2014) The reduction of Implant-Related Errors and Waste in total knee arthroplasty using a Novel, Computer Based, e.Label and Compatibility System. J Arthroplast. 10.1016/j.arth.2013.03.013 10.1016/j.arth.2013.03.013 23618749

[23] Epstein NE, Roberts R, Collins J (2015) Operative costs, reasons for operative waste, and vendor credit replacement in spinal surgery. Surg Neurol Int. 10.4103/2152-7806.156574 26005582 10.4103/2152-7806.156574 PMC4431048

[24] Pfefferle KJ, Dilisio MF, Patti B, Fening SD, Junko JT (2015) Transparency to Reduce Surgical Implant Waste. 10.4055/cios.2015.7.2.207. Clin Orthop Surg 10.4055/cios.2015.7.2.207 PMC4515461 26217467

[25] Soroceanu A, Robinson A, Canacari E, McGuire K (2011) Intra-operative waste in spine surgery: incidence, cost and effectiveness of an awareness program. Spine J. 10.1016/j.spinee.2011.08.172 10.1097/BRS.0b013e31822a58c1 21738100

[26] Zywiel MG, Ulrich SD, Suda AJ, Duncan JL, McGrath MS, Mont MA (2010) Incidence and cost of intraoperative waste of hip and knee arthroplasty implants. J Arthroplasty. 10.1016/j.arth.2009.03.005 19447003 10.1016/j.arth.2009.03.005

[27] Laurut T, Duran C, Pages A, Morin MC, Cavaignac E (2019) What is the cost burden of surgical implant waste? An analysis of surgical implant waste in an orthopedics and trauma surgery department of a French university hospital in 2016. Orthop Traumatol Surg Res. 10.1016/j.otsr.2019.06.010 31473131 10.1016/j.otsr.2019.06.010

[28] Payne A, Slover J, Inneh I, Hutzler L, Iorio R, Bosco JA 3rd (2015) Orthopedic Implant Waste: analysis and quantification. Am J Orthop (Belle Mead NJ) 44(12):554–560 26665242

[29] Huet S, Desclee de Maredsous R, Almeida M, Brischoux S, Marcheix PS (2022) The use of single-use ancillaries does not increase the cost of osteosynthesis in orthopaedic surgery: a case study of plate osteosynthesis for distal radius fractures. 10.1016/j.injury.2022.04.016. Injury 10.1016/j.injury.2022.04.016 35489821

[30] Kooner S, Hewison C, Sridharan S, Lui J, Matthewson G, Johal H, Clark M (2020) Waste and recycling among orthopedic subspecialties. Can J Surg. 10.1503/cjs.018018 32437094 10.1503/cjs.018018 PMC7828994

[31] Pegg M, Rawson R, Okere U (2022) Operating room waste management: a case study of primary hip operations at a leading National Health Service hospital in the United Kingdom. J Health Serv Res Policy. 10.1177/13558196221094488 35635489 10.1177/13558196221094488

[32] Rammelkamp Z, Dirnberger J, Johnson G, Waisbren S (2021) An audit of all Waste leaving the operating room: can the Surgical suite be more environmentally sustainable? World Med Health Policy. 10.1002/wmh3.397

[33] Southorn T, Norrish AR, Gardner K, Bax R (2013) Reducing the carbon footprint of the operating theatre: a multicentre quality improvement report. J Perioper Pract. 10.1177/175045891302300605 10.1177/175045891302300605 23909168

[34] Hennessy O, Diack M, Devitt A (2021) Screwing our environment: an analysis of orthopaedic implant related waste. Ir Med J 114(2):P266

[35] Baxter NB, Yoon AP, Chung KC (2021) Variability in the Use of Disposable Surgical supplies: a Surgeon Survey and Life Cycle Analysis. J Hand Surg. 10.1016/j.jhsa.2021.05.027 10.1016/j.jhsa.2021.05.027 34275683

[36] Streit JJ, Youssef A, Coale RM, Carpenter JE, Marcus RE (2013) Orthopaedic surgeons frequently underestimate the cost of Orthopaedic Implants. Clin Orthop Relat Research^®^. 10.1007/s11999-012-2757-x 10.1007/s11999-012-2757-x PMC3706639 23250855

[37] Santiesteban L, Hutzler L, Bosco JA 3rd, Robb W 3rd (2016) Wrong-site surgery in Orthopaedics: prevalence, risk factors, and strategies for Prevention. JBJS Rev 4. 10.2106/jbjs.Rvw.O.00030 10.2106/JBJS.RVW.O.00030 27490006

[38] Haene RA, Sandhu RS, Baxandall R (2009) Reading the Small print– labelling recommendations for Orthopaedic implants. Annals Royal Coll Surg Engl. 10.1308/003588409x432392 10.1308/003588409X432392 PMC2966240 19686615

[39] Knight DM, Wedge JH (2010) Marking the operative site: a lesson learned. CMAJ. 10.1503/cmaj.091860 20682732 10.1503/cmaj.091860 PMC2988567

[40] Haene RA, Sandhu RS, Baxandall R (2009) Reading the small print - labelling recommendations for orthopaedic implants. Ann R Coll Surg Engl. 10.1308/003588409x432392 19686615 10.1308/003588409X432392 PMC2966240

[41] Ledet EH, Liddle B, Kradinova K, Harper S (2018) Smart implants in orthopedic surgery, improving patient outcomes: a review. Innov Entrep Health. 10.2147/ieh.S133518 30246037 10.2147/IEH.S133518 PMC6145822

[42] Bell SW, Anthony I, Jones B, MacLean A, Rowe P, Blyth M (2016) Improved accuracy of component positioning with robotic-assisted unicompartmental knee arthroplasty: data from a prospective, randomized controlled study. J Bone Joint Surg Am. 10.2106/jbjs.15.00664 27098321 10.2106/JBJS.15.00664

