# Supplementary material for: Implant waste and associated costs in trauma and orthopaedic surgery: a systematic review
Source: Int Orthop. 2025 Jan 4;49(2):323–34. doi: 10.1007/s00264-024-06397-w (PMC11762414; doi:10.1007/s00264-024-06397-w)
Supplement: Supplementary file 1 — Supplementary Material 1 [file 264_2024_6397_MOESM1_ESM.docx]

**Supplementary material:**

Item A: Search terms used for PubMed

("hospital waste" OR "implant waste" OR "surgical waste" OR "operative waste" OR "unused" OR "unusable" OR "trash" OR “disposal” OR “dispose”) AND ("orthopedic" OR "orthopaedic" OR "orthopedic surgery" OR "orthopaedic surgery" OR "trauma surgery" OR "trauma" OR "T&O" OR "Fracture" OR "Fixation surgery" OR "Intramedullary nail" OR "Screw" OR "Plate" OR "ORIF" OR "Internal fixation" OR "Implant")

Item B: Search terms used for databases Embase and Scopus.

| **Search criteria** | **Search term** | **Number of results** |
| --- | --- | --- |
| 1 | exp hospital waste/ | 4,076 |
| 2 | "implant wast*".ab,ti. | 10 |
| 3 | "surgical wast*".ab,ti. | 164 |
| 4 | "surgical wast*".ab,ti. | 164 |
| 5 | "operati* wast*".ab,ti. | 100 |
| 6 | unused.ab,ti. | 5,729 |
| 7 | unusable.ab,ti. | 1,348 |
| 8 | trash.ab,ti. | 1,420 |
| 9 | dispose.ab,ti. | 3,229 |
| 10 | disposal.ab,ti. | 40,980 |
| 11 | exp orthopedic surgery/ | 589,766 |
| 12 | "orthop?edi*".ab,ti. | 143,907 |
| 13 | orthop?edic surgery.ab,ti. | 22,163 |
| 14 | trauma surgery.ab,ti. | 4,309 |
| 15 | trauma.ab,ti. | 362,177 |
| 16 | "Fractur*".ab,ti. | 366,959 |
| 17 | T&O.ab,ti. | 0 |
| 18 | Fixation surgery.ab,ti. | 553 |
| 19 | Intramedullary nail.ab,ti. | 3,761 |
| 20 | Screw.ab,ti. | 47,514 |
| 21 | Plate.ab,ti. | 157,039 |
| 22 | ORIF.ab,ti. | 3,329 |
| 23 | Internal fixation.ab,ti. | 25,618 |
| 24 | "Implant*".ab,ti. | 655,809 |
| 25 | 1 or 2 or 3 or 4 or 5 or 6 or 7 or 8 or 9 or 10 | 54,284 |
| 26 | 11 or 12 or 13 or 14 or 15 or 16 or 17 or 18 or 19 or 20 or 21 or 22 or 23 or 24 | 1,896,408 |
| 27 | 25 and 26 | 1,811 |
